# Supplementary figures and images for: The timing of transcription of RpoS-dependent genes varies across multiple stresses in Escherichia coli K-12
Source: mSystems. 2023 Aug 25;8(5):e00663-23. doi: 10.1128/msystems.00663-23 (PMC10654073; doi:10.1128/msystems.00663-23)

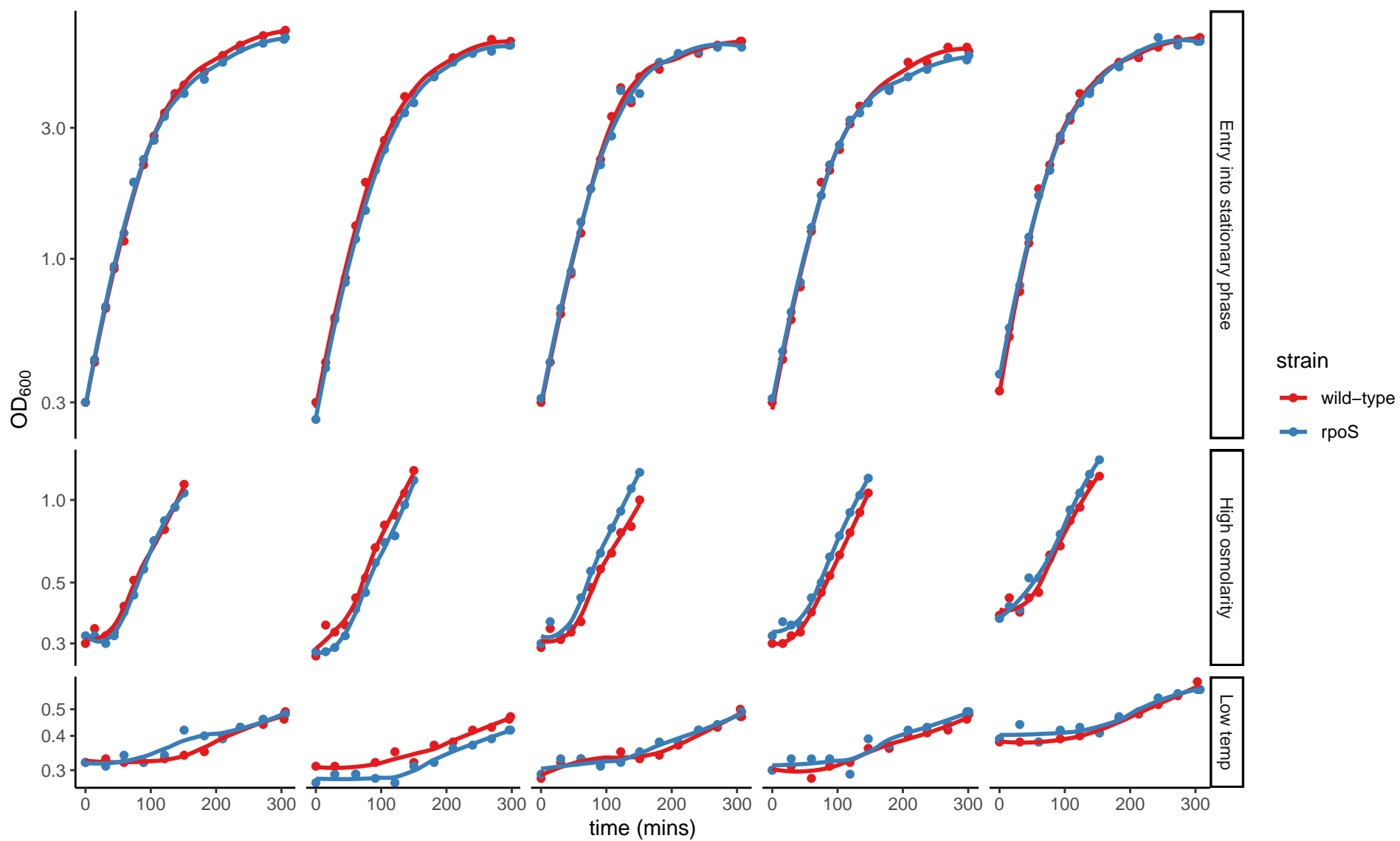

Supplement: Figure S1 — Growth curves of wild-type and ∆rpoS strains during the entry into stationary phase, high osmolarity, and low temperature. [file msystems.00663-23-s0001.pdf]

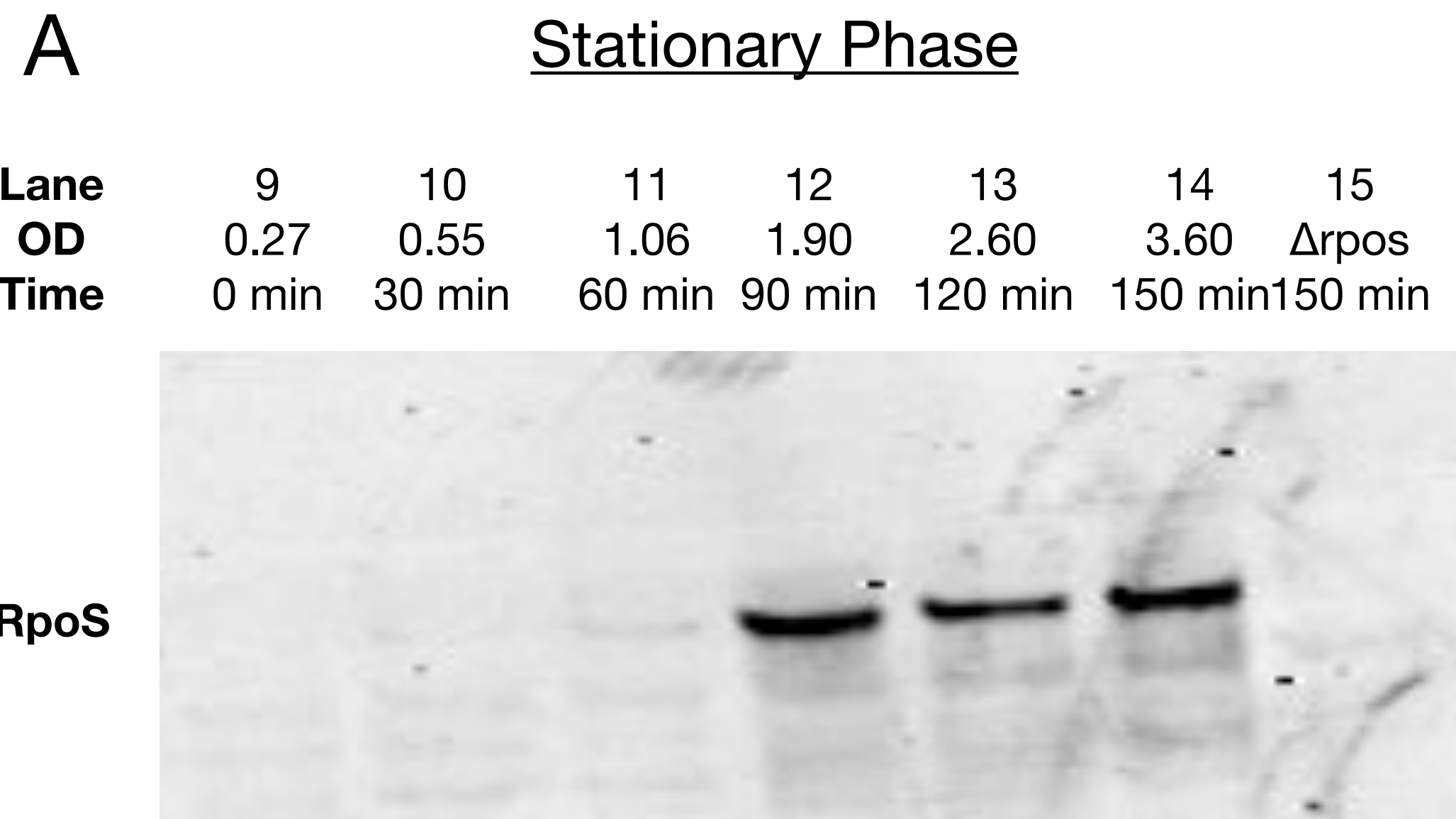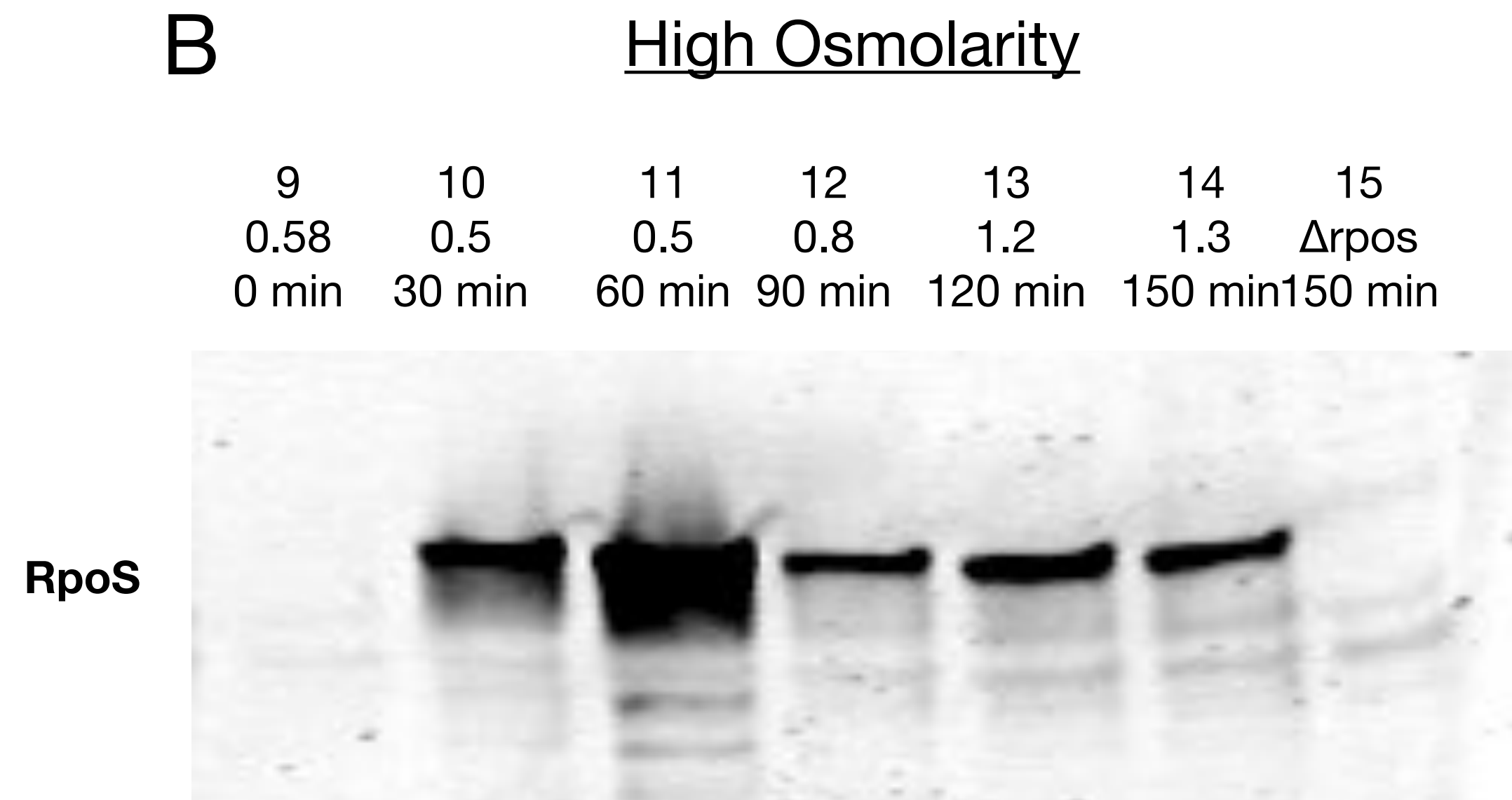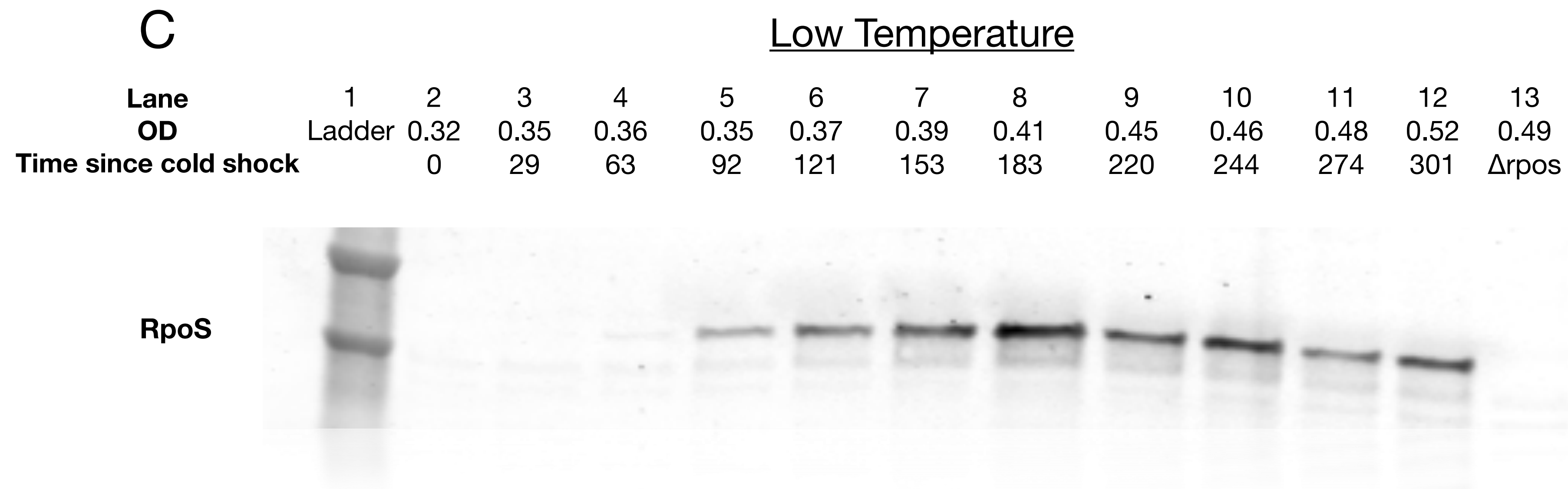

**Figure S2: Examples of western blots of RpoS levels across the three stresses.**

Supplement: Figure S2 — Examples of western blots of RpoS levels across the three stresses. [file msystems.00663-23-s0002.pdf]
